# ESF-EMBO Symposium: Antiviral Applications of RNA Interference

**DOI:** 10.1186/1742-4690-5-81

**Published:** 2008-09-18

**Authors:** Olivier ter Brake, Joost Haasnoot, Jens Kurreck, Ben Berkhout

**Affiliations:** 1 Laboratory of Experimental Virology, Department of Medical Microbiology, Center of Infection and Immunity Amsterdam (CINIMA), Academic Medical Center, University of Amsterdam, Meibergdreef 15, 1105 AZ Amsterdam, The Netherlands; 2 Institute of Industrial Genetics, University of Stuttgart, Allmandring 31, 70569 Stuttgart, Germany

## Introduction

The first ESF-EMBO symposium on "Applications of antiviral RNA interference (RNAi)" was held in the spring of 2008 (5–10 april) in Sant Feliu de Guixols at the Costa Brava in Spain. Some 60 participants from the fields of RNAi research and virology came together to present their latest findings on RNAi-virus interactions, as well as the progress in the development of RNAi-based antiviral therapeutics. One of the big topics concerned the role of RNAi in natural antiviral defence mechanisms in mammals [1-3]. In addition, new solutions to improve the efficacy and safety of RNAi-based antiviral drugs were presented. The combined expertise of researchers studying RNAi in plants, insects and mammalian systems greatly stimulated the overall discussion. The meeting was funded by the European Science Foundation (ESF) in partnership with the European Molecular Biology Organisation (EMBO).

## RNAi in gene regulation and antiviral responses

RNAi is a post transcriptional gene silencing mechanism that is triggered by double-stranded RNA (dsRNA). RNAi and RNAi-related mechanisms play essential roles in the regulation of cellular gene expression, as well as in innate antiviral immune responses. As such, the importance of RNAi in eukaryotic cell biology can hardly be overestimated. In addition to its natural functions, RNAi as a tool to specifically silence genes has in recent years revolutionized molecular biological research, and has provided new possibilities in drug design [4]. Despite the fact that these RNAi tools are now commonly used, still relatively little is known about the natural functions of RNAi. So far the role of RNAi in regulation of gene expression via endogenously expressed microRNAs (miRNAs) has received a lot of attention [5]. miRNAs are small non-coding RNAs that are expressed as long precursor RNAs (primary miRNAs) that are processed by the Drosha and Dicer enzymes into a stem loop precursor miRNA (pre-miRNA) and the mature miRNA (21–23 nucleotides), respectively. After the mature miRNA is loaded into the RNA-induced silencing complex (RISC, sometimes referred to as miRISC), the complex targets complementary sequences within the 3'UTR of a target messenger RNA, resulting in translational repression. It is currently estimated that expression of at least 30% of all human genes is regulated by miRNAs [6]. The exact criteria for target recognition are not clear. However, pairing of the 5' 7–8 nucleotides of the miRNA (seed region) to the 3' untranslated region of a target mRNA is in many cases sufficient to trigger translational inhibition [5-8].

The antiviral role of RNAi is well established in plants, insects and nematodes [9]. In these organisms virus infection results in the production of virus-specific siRNAs that target the viral RNA. These antiviral siRNAs arise from dsRNA replication intermediates but have also been shown to originate from sequences folding into extensive secondary structures [10]. A cellular RNA-dependent RNA polymerase is required for amplification of the siRNA signal and to trigger a potent antiviral RNAi response [11]. Plant and insect viruses counter the antiviral RNAi response by expressing RNAi or silencing suppressor factors. At this meeting it again became clear that there is still a lot of discussion about whether or not similar antiviral RNAi responses play a role in mammals [1]. Similar to plant and insect viruses several mammalian viruses have been shown to encode factors that can inhibit RNAi, suggesting repression by antiviral RNAi responses [12]. In addition, cellular miRNAs have been shown to target viral mRNAs [13-16]. However, so far virus specific siRNAs could not be detected in virus infected mammalian cells. Possibly, this is a technical issue. At the meeting it was suggested that new deep sequencing technology may provide the sensitive tool that is required to identify virus specific siRNAs in mammalian cells.

## RNAi technology

The first session of the meeting focused on RNAi technology. The most common strategies to induce RNAi are stable intracellular expression of short hairpin RNA (shRNA) or transient transfection of synthetic small interfering RNAs (siRNAs). **Mark Kay **from Stanford University discussed RNAi-based gene therapy approaches against virus-induced hepatitis using shRNAs. One of the problems of this approach is that the adeno associated virus vector used to deliver the shRNA-expression cassette can trigger an immune response. To solve this problem one could transiently suppress the immune response. However, a more elegant method to evade immunity is to select for less immunogenic vectors via capsid shuffling. This approach resulted in a 100.000 fold more effective vector. Another problem that was discussed was shRNA toxicity [17]. Previously, it was shown that overexpression of virus-specific shRNAs in liver caused lethality in mice by saturation of Exportin 5 (Exp5), thus interfering with export and maturation of endogenous microRNAs (miRNAs). New data was presented that also implicated Ago2, the slicer in the RNA-induced silencing complex (RISC), as a rate limiting factor. Combined overexpression of Ago2 and Exp5 reduced toxicity associated with shRNA overexpression and enhanced shRNA knockdown activity.

Focusing on the RNAi mechanism, Mark Kay and his co-workers also asked the question why miRNA targets are only functional in the 3'UTR of the mRNAs and not in the open reading frame (ORF). Data was presented indicating that miRNA translational inhibition is affected by the speed of the translating ribosome. miRNA target sequences within ORFs can in fact become functional when translation is slowed down, e.g. when a miRNA target site is preceded by rare codons. Earlier, Lytle and co-workers also showed functionality of miRNA targets in 5'-UTRs of reporter genes, and concluded that any position on a target RNA may be mechanistically sufficient to repress translation [18].

Besides the use of viral vector systems for intracellular expression of RNAi-inducers, synthetic siRNAs are also considered highly effective candidate therapeutics. Joachim Engels (Goethe University Frankfurt) gave some background information about the chemical synthesis of siRNAs. Developments like the 2'-acetoxyethyl (ACE) RNA chemistry and the incorporation of modified, especially cationic, nucleotides form the basis for the synthesis of highly stable effective siRNAs. Jorgen Kjems (University of Aarhus) discussed some of the latest developments in the use of modified siRNAs. One of the major problems with synthetic siRNAs is their low stability in serum. A comprehensive study was conducted with many different chemistries at the 2'O ribose position such as aminoethyl and guanidinoethyl [19], and it was shown that siRNA half life and efficacy can be greatly enhanced by introducing modifications at specific positions both in the passenger and the guide strand of the siRNA. Off-target effects caused by the incorporation of the passenger strand in RISC were effectively avoided by design of a nicked passenger strand in the so called small internally segmented interfering RNA (sisiRNA) design. Furthermore, off-target effects could be avoided by incorporation of specific modifications in the guide strand of the siRNA. In addition, Kjems focussed on siRNA delivery systems and showed that nanoparticles based on chitosan were highly effective for siRNA delivery, particularly in the lungs.

An interesting novel technique termed RNAu was presented by Puri Fortes (University of Navarra) [20]. RNAu is based on expression of U1 small nuclear RNA (snRNA) of which the 5' nucleotides 2–11 are modified to base-pair with a 10 nucleotide target within the 3' terminal exon of a gene of interest. Binding of the modified U1 snRNA inhibits polyadenylation, resulting in degradation of the transcript and gene knockdown. The U1 snRNA mechanism tolerates a single mismatch at positions 1, 2, 9 and 10, the central 6 nucleotides require perfect base-pairing but do allow a single G-U base-pair. The presence of multiple target sites within the 3' exon enhances inhibition, and a knockdown of gene expression of up to 700-fold can be achieved. Interestingly, when combined with RNAi, additive or even synergistic inhibition was obtained.

## Plants viruses and RNAi

Thomas Hohn (University of Basel) introduced the mechanism of RNAi in plants and its interaction with viruses. Plants use RNAi as an antiviral defence in which viral replication intermediates in the form of dsRNA are processed by the Dicer-like enzyme (DCL) [9]. Furthermore, plants can amplify the RNAi effect using RNA-dependent RNA polymerase (RdRP) and siRNAs as primers. The RNAi machinery in plants is rather complex with four DCL enzymes: DCL1 processes primary-miRNA (pri-miRNAs) with different product sizes depending on the substrate, DCL2-4 process dsRNA. DCL2 can compensate for deficiencies in the other DCL enzymes and yields a 22-nucleotide (nt) product, DCL3 and 4 produce 24 and 21-nt siRNAs, respectively. RNAi in plants can be triggered by DNA viruses and RNA viruses. For instance, Hohn showed that two DNA viruses, the Cabbage leaf curl virus (CaLCuV) and the Cauliflower mosaic virus (CaMV), triggered the synthesis of 21, 22 and 24-nt siRNAs, and the cytoplasmic RNA tobamovirus Oilseed rape mosaic virus (ORMV) triggered predominantly 21-nt siRNAs. Since the RNAi machinery in plants can act as a potent antiviral response, viruses in turn have evolved RNA silencing suppressors (RSS) as a countermeasure. For instance, the p19 protein from Tombusvirus can bind and neutralize siRNAs. Interestingly, the AC2 protein from Mungbean yellow mosaic virus-Vigna (MYMV) is not an RNAi suppressor itself, but apparently triggers the activation of an endogenous RSS activity.

Björn Krenz (University of Stuttgart) reported on the Abutilon Mosaic Virus, which was engineered as a versatile vector to deliver genes in to plants. It was subsequently employed to silence phytoene desaturase in Nicotiana benthamiana, demonstrating that this viral vector is a valuable tool for functional studies. Juan Antonio García (CNB-CSIC, Madrid) presented work on the cucumber vein yellowing Virus (CVYV), which is a member of the potyviridae. Remarkably, CVYV does not encode the silencing suppressor HCPro that is typical for potyviridae, but instead produces the P1a-b protein that is proteolytically processed into P1a and P1b instead of a single P1 protein. P1b is a serine protease that accumulates in infected plants and functions as an RSS. It contains a Zn-finger and LXKA basic motif, which are both required for RSS function. P1b binds siRNAs but also endogenous miRNAs, which affects the miRNA expression pattern of the host cell. In the plum pox virus, HCPro could be replaced by P1b, adding further proof that P1b is an RSS.

Kirsi Lehto (University of Turku) presented data on plant virus encoded RSS factors and their role in virus-induced disease. RSS genes derived from six virus genera were transformed into Nicotiana benthamiana and N. tabacum plants. Depending on the species of the host plant the RNA silencing suppressors caused different disease phenotypes. In addition, the suppressors demonstrated different effects on crucifer-infecting Tobamovirus (crTMV) infections. Apparently, these suppressors act at different levels in the RNAi pathway, and interfere with miRNA function to variable degrees.

Olivier Voinnet (Institute de Biologie Moléculaire des Plantes, Strasbourg) showed that the interaction between host and pathogen is more complicated than simple defence and counterdefence mechanisms. Arabidopsis encodes for 10 different Ago genes, Ago1 minus plants are hypersensitive to viruses indicating that Ago1 is involved in antiviral responses. Previously, Ago1 was shown to act within the miRNA pathway. Thus, miRNA and antiviral pathways appear to converge. In addition to RNA silencing, resistance (R) genes are also involved in blocking virus replication in plants. These genes encode receptors that detect pathogens and activate strong defences similar to pattern-recognition receptors in mammals [21]. It is becoming clear that genes involved in RNAi are in fact R genes that regulate the hypersensitive response (HR). HR causes apoptosis of the local region surrounding the infection thus preventing further viral spread. There is also evidence that HR factors are part of RISC. Although the antiviral function of RNAi in mammals is still debated, Olivier Voinnet extended the function of RNAi in plants to a defence against bacterial pathogens [22,23]. Specific plant miRNAs are induced in response to bacterial pathogens that are detected via the flagellin receptor. Similar to viruses, bacteria also encode specific factors that are translocated to the plant cells to block the miRNA pathway. These effectors were identified and found to affect processing of Ago1. In this way, viral and bacterial infections can join forces and benefit from each others presence by a severe attack on the RNAi defence mechanism.

## Drosophila and innate antiviral responses

In this session the focus was on RNAi mechanisms in Drosophila and their interaction with viruses. Drosophila encodes two Dicer enzymes, Dcr1 is involved in miRNA processing and Dcr2 processes dsRNA into siRNAs. The RNAi mechanism acts as a potent innate response against viral infection. Ronald van Rij (Radboud University Nijmegen) presented data highlighting the antiviral role of RNAi in insect cells by showing that Ago2-minus Drosophila melanogaster exhibit increased susceptibility to Drosophila C virus (DCV) infection and that the virus encodes an RSS. Using Sindbis virus, which can infect both mammalian and insect cells, it was shown that knockdown of Ago2 and Dcr2 in insect cells increased virus production, whereas knockout of Dcr in mouse cells had no effect. These results suggest that RNAi has no role in the mammalian antiviral defence against Sindbis virus.

Jean-Luc Imler (Université de Strasbourg) also showed that RNAi is an antiviral response mechanism in insects. He demonstrated that Dcr2-minus cells are more sensitive to Flock house virus (FHV) and that the B2 protein is an RSS that binds dsRNA. More importantly, he presented interesting work suggesting that the virus-specific RNAi response triggers a secondary antiviral response involving JAK/STAT signalling and the production of cytokines. A key factor in this cellular response pathway is Vago, whose induction is also suppressed by the FHV B2 protein, indicating that dsRNA triggers the inducible antiviral response.

Carla Saleh (Institute Pasteur, Paris) presented data on the spread of the RNAi signal to neighboring cells. In plants, systemic spread of the antiviral RNAi signal is important for viral clearance. Similar mechanisms were thus far not observed in flies. Insect cells do not take up siRNAs, but they can take up large dsRNA molecules that subsequently induce RNAi. Cellular factors involved in this RNA-uptake were identified and knockout mutant flies were shown to be hypersensitive to viral infection by Sindbis and DCV. Virus-induced cell lysis results in release and spread of virus-specific dsRNA molecules that are taken up by uninfected surrounding cells, thus generating an antiviral state.

## Interactions between mammalian viruses and cellular RNAi mechanisms

Recently, it has become clear that mammalian viruses interact with components of the host RNAi machinery. Viruses can express miRNAs to regulate the expression of cellular genes, or viral gene expression may be activated or repressed by cellular miRNAs. In addition, several viruses encode suppressors of RNAi. A separate session was dedicated to these complex interactions between viruses and the RNAi machinery.

Goran Akusjarvi (Uppsala University) presented data on how adenovirus interacts with the RNAi/miRNA pathways. He showed that the structured non-coding virus-associated RNAs (VA RNA I and II) are processed by Dicer and incorporated into RISC. Although only 2–5% of the total amount of the VA RNAs is diced, up to 80% of all RISC complexes contain VA-derived si/miRNAs late in infection [24]. Of these, ~80% stem from VA RNAII, which is expressed at much lower levels than VA RNAI. Besides this VA RNAII bias, there also appears to be a strand bias for incorporation into RISC. Data was presented that this bias may arise from two different transcription initiation sites that are used during VA RNA expression. Puri Fortes (University of Navarra) presented data that blocking of the adenoviral VA miRNAs results in a decrease in viral titer, suggesting that VA miRNAs control the expression of genes whose expression affects adenovirus production. This group has also identified several putative targets for these miRNAs using a combination of bioinformatic approaches and microarray analysis. How these targets affect the viral cycle remains to be established.

## Does RNAi play a role in antiviral immune responses in mammals?

One of the most fiercely discussed issues during the meeting was the question whether or not RNAi has a role in antiviral mechanisms in mammals. On the one hand, it has been shown that several mammalian viruses encode RSS functions, implying that the virus must have evolved this functionality in order to suppress RNAi [12]. On the other hand, virus-specific siRNAs could thus far not be detected in virus-infected mammalian cells, which is unlike the situation in plant and insect cells. Bryan Cullen (Duke University) started the discussion by summarizing data that do not support a role for antiviral RNAi responses in mammals. For example, long dsRNA induces the interferon (IFN) response in mammalian cells whereas these molecules trigger a potent and specific RNAi response in plants and insects. Furthermore, human immunodeficiency virus type 1 (HIV-1) infection does not result in the production of virus-specific siRNAs. The reported RNAi suppression activity of the HIV-1 Tat protein and the primate foamy virus type 1 (PVF-1) Tas protein was claimed to result from promoter activation rather than RNAi suppression. For example, the Tat-induced increase in expression of a shRNA silenced reporter would result from activation of the promoter controlling firefly expression instead of blocking the shRNA-induced RNAi response. Cullen concluded that mammalian viruses neither induce nor repress siRNAs because there is no need to do so. Instead, mammalian viruses use the RNAi pathway for their own benefit by expression of virus-encoded miRNAs that target cellular mRNAs. Cullen showed that the Herpes simplex virus type 1 (HSV-1) expresses several miRNAs from the LAT-gene that target viral immediate early mRNAs. These miRNAs did not include the miR-LAT that was previously reported. Interestingly, the viral miRNAs trigger inhibition of translation despite the fact that the mRNA target is located within the ORF sequences.

The discussion on the role of RNAi in mammals was continued by Kuan-Teh Jeang (National Institutes of Health, USA) who summarized literature data that favour the antiviral role of RNAi in mammals [25]. In addition, he presented preliminary results from deep-sequencing analysis of small RNAs from HIV-1 infected cells. In total, 163 clones of several small virus-specific RNAs were detected. It is currently unclear whether these are incorporated into RISC and thus represent antiviral siRNAs. It also needs to be excluded that these small RNAs merely represent degraded RNA, although the discrete size range of these RNAs suggests that this is not the case. Besides these de novo produced virus specific small RNAs, several groups have recently shown that cellular miRNAs can also target and inhibit the expression of viral mRNAs. However, the physiological significance of such a mechanism is debated because it appears paradoxical for the virus to retain functional miRNA target sites in their RNA genome. Possibly, the virus benefits from downregulation by miRNAs.

Fatah Kashanchi (George Washington University) presented data suggesting that the TAR hairpin at the 5' end of HIV-1 transcripts is recognized by Dicer and processed into functional miRNAs. The amount of TAR miRNAs produced seems to vary significantly between different HIV-1 infected cell lines. In the absence of Tat protein, these short transcripts appear to be extremely abundant both in cell lines and latent primary infected cells. This suggests that perhaps the TAR miRNA is involved in transcriptional silencing of the integrated proviral DNA genome, thereby contributing to latency. Finally, in a recent collaboration with the group of Zvi Bentwich (Rosetta Genomincs Ltd., Israel), they have been able to clone the TAR miRNAs from infected cells.

Monsef Benkirane (Institute de Genetique Humaine, Montpellier) showed that knockdown of Drosha, Dicer and DGCR8 in mammalian cells resulted in increased HIV-1 production, which was linked to the previously reported role of miRNAs in maintenance of HIV-1 latency [13]. However, Anne Gatignol (McGill University, Montreal) showed that knockdown of TRBP and Dicer resulted in a decrease in virus production. These results appear to be contradictory but may arise from specific differences in experimental set up. Enhancers and repressors of virus replication may both be regulated by miRNAs. Knockdown of the RNAi pathway may therefore go either way. Previously, Huang and co-workers showed that cellular miRNAs are involved in the control/maintenance of latency [13]. It was suggested that these miRNAs may represent new antiviral drug targets. Inhibition of these specific latency miRNAs would result in activation of latent virus reservoirs that are normally difficult to target with highly active antiretroviral therapy (HAART). Activation of the HIV-1 reservoirs would allow recognition and elimination of all infected cells by the immune system.

Joost Haasnoot (University of Amsterdam) also presented data on the interplay of cellular miRNAs and HIV-1 replication. miRNA expression profiles were studied in HIV-1 infected T-cells using a quantitative RT-PCR approach. In HIV-1 producing cells, 11 out of a total of 293 studied miRNAs were significantly affected. A bioinformatics analysis indicated that 8 of these 11 miRNAs have potential target sites within the HIV-1 genome. These miRNAs add to the current list of candidate miRNAs that target HIV-1. Interestingly, these new targets cluster to specific regions of the HIV-1 genome, suggesting a positive selection during virus evolution. Anne Gatignol addressed whether viruses inhibit the endogenous RNA silencing pathways, e.g. by means of a suppressor protein. Whereas HIV-1 did not inhibit RNAi-mediated knockdown in cells transfected with exogenous shRNAs, such an inhibition was exerted by the virus on cell endogenous miRNAs that target perfecty complementary sites in a reporter gene.

## HIV-1 RNAi therapeutics

A major problem with antiviral approaches against HIV-1 is the emergence of escape variants. Similar to the emergence of drug resistant mutations, RNAi resistant mutations have also been described [4]. Thus, for the development of effective RNAi-based therapies against escape-prone viruses, the main objective is to effectively suppress virus replication while preventing the selection of resistant variants. In case of HIV-1 this is further complicated by the large heterogeneity of viral sequences within a patient. Miguel Angel Martinez (irsiCAixa Foundation, Barcelona) described two approaches aimed at preventing viral escape. First, one could counteract escape mutations against a specific siRNA by including second generation siRNAs that are directed against these specific mutants. In addition, one could also inhibit the virus with multiple siRNAs generated in vitro from Dicer-cleaved long dsRNA.

Karin Metzner (University of Erlangen) addressed the problem of HIV-1 resistance against regular antiviral drugs. It was proposed to use RNAi to specifically suppress these escape variants. Combining 3TC, a nucleoside Reverse Transcriptase inhibitor, with an siRNA directed against the most common 3TC-resistance mutation (Met184Val), proved to be effective in cell culture infections. Targeting essential cellular co-factors could be a valid approach to avoid RNAi resistance but also a way of defining new therapeutic targets. Eduardo Pauls (irsiCaixa Foundation, Barcelona) showed that targeting of αV integrin and β5 integrin with siRNAs could inhibit HIV-1 replication. This inhibition was not at the level of virus entry, reverse transcription or integration but appeared to block transcription from the HIV-1 long terminal repeat promoter. However, siRNAs were used in all of the above-mentioned approaches, and siRNA delivery in patients is still a major bottleneck.

Olivier ter Brake (University of Amsterdam) presented results on the development of an RNAi-based gene therapy for HIV-1. A single treatment with a lentiviral vector expressing a single shRNA results in stable induction of RNAi. In a combinatorial approach, four antiviral shRNAs were expressed from a single lentiviral vector. In a T cell line containing a single vector copy per cell, HIV-1 replication could be effectively controlled for up to 40 days, while escape mutants emerged in control single shRNA cell lines. This result highlights the therapeutic potential of such an approach. However, safety aspects still require intensive investigation. A pilot study was performed in a humanized mouse model in which Rag2^-/-^γ_c_^-/- ^irradiated newborn mice are engrafted with shRNA-transduced human haematopoietic stem cells. Development of the immune system was not affected by constitutive shRNA expression, although a slightly reduced engraftment efficiency of the transduced cells was observed. Furthermore, sequence-specific inhibition of HIV-1 replication was demonstrated in CD4+ T cells from this mouse [26].

## Combining antiviral RNAi with immune stimulation

Hepatitis C virus (HCV) virus infection is a major cause of chronic liver disease with nearly 200 million carriers worldwide. The current standard treatment with pegylated-interferon-alpha (IFN-α) administered in combination with Ribavirin is only effective in half of the patients, prompting the need for alternative therapies. RNAi represents an attractive new approach against HCV, allowing knock-down of viral RNA or host factors involved in the virus life cycle. Based on their distinct antiviral mechanism, Qiuwei Pan (Erasmus University, Rotterdam) proposed that combining lentiviral vector mediated RNAi with IFN-α treatment may avoid therapeutic resistance and exhibit enhanced antiviral activity. However, there is some concern about a potential negative effect of IFN-α on vector transduction, but such an effect was not observed. Gunter Hartman (University of Bonn) presented his research at the interface of RNAi and interferon responses. Most researchers try to avoid siRNA side-effects. Instead, Hartman proposed to design siRNA specifically for immunorecognition and to use this additional activity for therapy. Such siRNA not only induce RNAi, but also TLR7 and RIG-I by inclusion of appropriate TLR7 motifs (5'-GUCCUUCAA-3', 5'-UGUGU-3' and derivatives thereof [27,28]), and 5'-triphosphates. Such an approach can be advantageous against viral infections and cancer.

## Cocksackie B3 and other viruses

Jens Kurreck (University of Stuttgart) presented data on RNAi-mediated inhibition of Coxsackie B3 virus (CoxB3). Using reporter constructs and virus he showed that only the plus-stranded RNAs can be targeted by the siRNAs. In addition, Kurreck showed that it is difficult to induce efficient RNAi knockdown when viral sequences are targeted that have complex RNA secondary structures.

Rainer Wessely (Munich University of Technology) gave an overview of CoxB3 involved in viral heart disease. siRNAs against CoxB3 were effective both *in vitro *and in an *in vivo *mouse model, yielding a 2–3 log reduction in virus replication. However, virus resistance was observed already after the first infection cycle, indicating that combinatorial RNAi approaches are required for effective and durable suppression. In an alternative approach, Sandra Pinkert (Charité, Berlin) demonstrated that CoxB3 can efficiently be inhibited in neonatal rat cardiomyocytes by vector mediated delivery of shRNA expression cassettes against the virus genome or its receptor, the coxsackievirus-adenovirus receptor (CAR). A soluble variant of CAR fused to the Fc domain of a human immunoglobulin had an even more potent antiviral effect suggesting that it might be worth to combine the different approaches.

**Carolyn Coyne **(University of Pittsburgh) uses RNAi to investigate entry of enteroviruses into polarized endothelial cells. Recently, she used a large scale screen to identify genes involved in entry of CoxB3 and poliovirus. One of the hits, the Yes kinase was characterized in more detail by low molecular weight inhibitors and its knockdown or inhibition was found to prevent entry of CoxB3 (but not of poliovirus) into human bone marrow endothelial cells.

Alexander Karlas (Max-Planck-Institute for Infection Biology, Berlin) reported on the use of RNAi against influenza virus A. siRNAs modified with locked nucleic acids (LNA) and delivered by chitosan were found to be efficient in a mouse influenza model. In order to identify host factors on which the virus depends large scale screens were performed and a large number of factors from the spliceosome were among the hits.

## Towards clinical applications

Jörg Kaufmann (Silence Therapeutics AG, Berlin) presented data on Atu027, an anti-cancer siRNA delivered systemically for the treatment of gastrointestinal cancer. Although not an antiviral RNAi approach, this presentation nicely listed the challenges of the clinical development of RNAi therapeutics. First of all, a formulation was developed, Atuplex, which consists of liposomes of ~120 nm containing a cationic lipid and a helper-lipid PEG-lipid, in which the siRNA is incorporated, a blunt ended 23-mer with 2'-O-methyl modification for stabilisation. The complex could be lyophilized and stored long-term at 4°C without significant loss of efficacy, an important requirement for clinical development. Furthermore, biodistribution, toxicology and efficacy studies were conducted in various animal models. The siRNA was found mostly in the endothelial cells of the lung but did not penetrate the tumor. No cytokines were induced, indicating that siRNA administration is safe. Furthermore, metastasis was reduced in a prostate cancer model. Combined, the data showed that Atu027 is effective and safe. Currently, Silence Therapeutics is preparing for a phase I clinical trial that is expected to start this year.

## Concluding remarks

In light of the new data presented at this meeting it is clearly too early to close the door on an antiviral function of RNAi in mammals. Instead, data in favour of an antiviral role of RNAi in mammals are accumulating. In addition, viruses and the cellular RNAi machinery interact in multiple different ways. This meeting has shown that both fundamental research on RNAi and viruses and the applications of RNAi technology are developing fast. An important discussion point during the meeting was about the future for RNAi therapeutics [29]. RNAi can be very potent and specific, underscoring the great potential of this mechanism. However, increasing concerns about toxicity and off-target effects have tempered these initial expectation for a rapid introduction of RNAi-based drugs in the clinic. Despite these concerns, pharmaceutic companies are investing in the further development of RNAi-based therapeutics. Currently, it is safe to say that we have only a limited understanding of the RNAi pathway and its functions. A more thorough understanding will contribute to the fine-tuning of RNAi-based drugs such that safe and effective RNAi based therapeutics can be developed.

## References

[B1] Cullen BR (2006). Is RNA interference involved in intrinsic antiviral immunity in mammals?. Nat Immunol.

[B2] de Vries W, Haasnoot J, van dV, van Montfort T, Zorgdrager F, Paxton W, Cornelissen M, van Kuppeveld F, de Haan P, Berkhout B (2008). Increased virus replication in mammalian cells by blocking intracellular innate defense responses. Gene Ther.

[B3] Yeung ML, Benkirane M, Jeang KT (2007). Small non-coding RNAs, mammalian cells, and viruses: regulatory interactions?. Retrovirology.

[B4] Haasnoot J, Westerhout EM, Berkhout B (2007). RNA interference against viruses: strike and counterstrike. Nat Biotechnol.

[B5] Grimson A, Farh KK, Johnston WK, Garrett-Engele P, Lim LP, Bartel DP (2007). MicroRNA targeting specificity in mammals: determinants beyond seed pairing. Mol Cell.

[B6] Krek A, Grun D, Poy MN, Wolf R, Rosenberg L, Epstein EJ, MacMenamin P, da PI, Gunsalus KC, Stoffel M, Rajewsky N (2005). Combinatorial microRNA target predictions. Nat Genet.

[B7] Brennecke J, Stark A, Russell RB, Cohen SM (2005). Principles of microRNA-target recognition. PLoS Biol.

[B8] Lewis BP, Shih IH, Jones-Rhoades MW, Bartel DP, Burge CB (2003). Prediction of mammalian microRNA targets. Cell.

[B9] Ding SW, Voinnet O (2007). Antiviral immunity directed by small RNAs. Cell.

[B10] Molnar A, Csorba T, Lakatos L, Varallyay E, Lacomme C, Burgyan J (2005). Plant virus-derived small interfering RNAs originate predominantly from highly structured single-stranded viral RNAs. J Virol.

[B11] Wassenegger M, Krczal G (2006). Nomenclature and functions of RNA-directed RNA polymerases. Trends Plant Sci.

[B12] de Vries W, Berkhout B (2008). RNAi suppressors encoded by pathogenic human viruses. Int J Biochem Cell Biol.

[B13] Huang J, Wang F, Argyris E, Chen K, Liang Z, Tian H, Huang W, Squires K, Verlinghieri G, Zhang H (2007). Cellular microRNAs contribute to HIV-1 latency in resting primary CD4(+) T lymphocytes. Nat Med.

[B14] Lecellier CH, Dunoyer P, Arar K, Lehmann-Che J, Eyquem S, Himber C, Saib A, Voinnet O (2005). A cellular microRNA mediates antiviral defense in human cells. Science.

[B15] Otsuka M, Jing Q, Georgel P, New L, Chen J, Mols J, Kang YJ, Jiang Z, Du X, Cook R, Das SC, Pattnaik AK, Beutler B, Han J (2007). Hypersusceptibility to vesicular stomatitis virus infection in Dicer1-deficient mice is due to impaired miR24 and miR93 expression. Immunity.

[B16] Triboulet R, Mari B, Lin YL, Chable-Bessia C, Bennasser Y, Lebrigand K, Cardinaud B, Maurin T, Barbry P, Baillat V, Reynes J, Corbeau P, Jeang KT, Benkirane M (2007). Suppression of microRNA-silencing pathway by HIV-1 during virus replication. Science.

[B17] Grimm D, Streetz KL, Jopling CL, Storm TA, Pandey K, Davis CR, Marion P, Salazar F, Kay MA (2006). Fatality in mice due to oversaturation of cellular microRNA/short hairpin RNA pathways. Nature.

[B18] Lytle JR, Yario TA, Steitz JA (2007). Target mRNAs are repressed as efficiently by microRNA-binding sites in the 5' UTR as in the 3' UTR. Proc Natl Acad Sci USA.

[B19] Odadzic D, Bramsen JB, Smicius R, Bus C, Kjems J, Engels JW (2008). Synthesis of 2'-O-modified adenosine building blocks and application for RNA interference. Bioorg Med Chem.

[B20] Abad X, Vera M, Jung SP, Oswald E, Romero I, Amin V, Fortes P, Gunderson SI (2008). Requirements for gene silencing mediated by U1 snRNA binding to a target sequence. Nucleic Acids Res.

[B21] Bent AF, Mackey D (2007). Elicitors, effectors, and R genes: the new paradigm and a lifetime supply of questions. Annu Rev Phytopathol.

[B22] Navarro L, Dunoyer P, Jay F, Arnold B, Dharmasiri N, Estelle M, Voinnet O, Jones JD (2006). A plant miRNA contributes to antibacterial resistance by repressing auxin signaling. Science.

[B23] Navarro L, Jay F, Nomura K, He SY, Voinnet O (2008). Suppression of the microRNA pathway by bacterial effector proteins. Science.

[B24] Xu N, Segerman B, Zhou X, Akusjarvi G (2007). Adenovirus virus-associated RNAII-derived small RNAs are efficiently incorporated into the rna-induced silencing complex and associate with polyribosomes. J Virol.

[B25] Grassmann R, Jeang KT (2008). The roles of microRNAs in mammalian virus infection. Biochim Biophys Acta.

[B26] Ter Brake O, Legrand N, von Eije KJ, Centlivre M, Spits H, Weijer K, Blom B, Berkhout B (2008). Evaluation of safety and efficacy of RNAi against HIV-1 in the human immune system (Rag-2(-/-)(c)(-/-)) mouse model. Gene Ther.

[B27] Judge AD, Sood V, Shaw JR, Fang D, McClintock K, Maclachlan I (2005). Sequence-dependent stimulation of the mammalian innate immune response by synthetic siRNA. Nat Biotechnol.

[B28] Hornung V, Guenthner-Biller M, Bourquin C, Ablasser A, Schlee M, Uematsu S, Noronha A, Manoharan M, Akira S, de FA, Endres S, Hartmann G (2005). Sequence-specific potent induction of IFN-alpha by short interfering RNA in plasmacytoid dendritic cells through TLR7. Nat Med.

[B29] Aagaard L, Rossi JJ (2007). RNAi therapeutics: principles, prospects and challenges. Adv Drug Deliv Rev.

